# Investigating the role of matrix habitat use in determining avian area‐sensitivity

**DOI:** 10.1002/ece3.6810

**Published:** 2020-11-13

**Authors:** H. Patrick Roberts, David I. King

**Affiliations:** ^1^ Department of Environmental Conservation University of Massachusetts Amherst MA USA; ^2^ U.S. Forest Service Northern Research Station University of Massachusetts Amherst MA USA

**Keywords:** area‐sensitivity, edge avoidance, matrix habitat use, *Setophagadiscolor*, *Setophagapensylvanica*, territory

## Abstract

The absence of some species from small habitat patches has long posed a challenge for conservationists, yet the underlying mechanisms that cause this “area‐sensitivity” remain poorly understood. Capacity of a species to extend their activities into the surrounding matrix habitat represents one potential determinant of area‐sensitivity. Species may be able to occupy smaller patches if they can utilize matrix habitat beyond patch boundaries, whereas area‐sensitive species may be restricted to larger patches due to their inability to utilize the surrounding matrix. We investigated the potential role of matrix utilization in determining area‐sensitivity by mapping the movements of two shrubland‐obligate passerines with contrasting patch area requirements in shrub‐dominated forest openings ranging in area by nearly an order of magnitude. Our findings were consistent with our predictions; the less area‐sensitive chestnut‐sided warbler (*Setophaga pensylvanica*) exhibited greater use of matrix habitat than the highly area‐sensitive prairie warbler (*S. discolor*). Furthermore, chestnut‐sided warblers that occupied smaller openings used mature forest more than conspecifics in larger patches, yet forest use by prairie warblers was unrelated to opening size. Chestnut‐sided warblers foraged as frequently in mature forest as within shrubland, whereas prairie warblers foraged significantly more in openings compared to forest. The findings of this study suggest that the ability or inclination of a species to utilize surrounding matrix habitat explains at least some of the observed variation in area‐sensitivity in songbirds and potentially other taxa.

## Graphical abstract

We investigated the role of matrix use in determining area‐sensitivity by mapping the movements of two shrubland‐obligate passerines with contrasting patch area requirements. The less area‐sensitive chestnut‐sided warbler (*Setophaga pensylvanica*) exhibited greater use of matrix habitat than the highly area‐sensitive prairie warbler (*S. discolor*). Furthermore, chestnut‐sided warblers that occupied smaller openings used mature forest more than conspecifics in larger patches, yet forest use by prairie warblers was unrelated to opening size. The findings of this study suggest that the ability or inclination of a species to utilize surrounding matrix habitat explains at least to some of the observed variation in area‐sensitivity in songbirds and potentially other taxa.

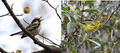

## 1 INTRODUCTION

Area‐sensitive species—those that are scarce or absent from small patches of habitat (Ambuel & Temple, 1983; Freemark & Collins, 1992; Robbins et al., 1989)—are often considered of conservation concern because they are typically the first to be locally extirpated when natural landscapes become fragmented (Bayard & Elphick, 2010; Bender et al., 1998). Considerable effort has been directed toward identifying area‐sensitive species (Connor et al., 2000) and characterizing their relative sensitivity by identifying respective area requirements (Roberts & King, 2017; Shake et al., 2012; Winter et al., 2006); however, the underlying mechanisms that cause area‐sensitivity in different taxa remain poorly understood (Lehnen & Rodewald, 2009; Ribic et al., 2009). In a review of how area‐sensitivity is studied in birds, Bayard and Elphick (2010) argued that area‐sensitivity is not necessarily an aversion to patch size itself, but rather a collection of interacting effects that are correlated with area. Factors that have been proposed include landscape context (Martensen et al., 2008), patch isolation, microhabitat characteristics (Ewers & Didham, 2006), food availability (Burke & Nol, 1998; Zanette et al., 2000), and conspecific attraction (Bourque & Desrochers, 2006; Fletcher, 2009).

Another explanation for area‐sensitivity in birds that has received considerable attention is the influence of edge effects (e.g.,Fletcher, Ries, Battin, & Chalfoun, 2007). Many bird species appear to avoid edges—perhaps due to increased nest predation risk (e.g., King & Byers., 2002; Shake et al., 2011) or other factors—and thus, may avoid small patches due to inherent proximity of edge (Banks‐Leite et al., 2010). Until recently, birds that nest in shrubland habitats were often broadly labeled edge specialists, but Schlossberg and King (2008) reported that at least eight shrubland‐associated species were significantly more abundant further from edges, suggesting that many shrubland species may in fact be edge‐averse. However, Lehnen and Rodewald (2013) showed, through a telemetry‐based study of the area‐sensitive (Shake et al., 2012) yellow‐breasted chat (*Icteria virens*), that reduced abundance near edges is more likely the result of passive displacement (i.e., fewer territories are placed near edges than the center of patches because territories cannot extend into the surrounding matrix [King et al., 1997, Fletcher & Koford, 2003]) rather than active edge avoidance. This supports the contention that area‐sensitivity may not be driven by edge avoidance, but rather the inability or lower capacity of area‐sensitive species to make use of the surrounding habitat matrix (Henle et al., 2004).

The view that area‐sensitivity is a function of a species' avoidance of, or inability to occupy, the matrix habitat is consistent with the view that territory size is a determinant of a given species’ minimum patch size requirements, and thus, patches of habitat must be large enough to fit an individual bird's territory in order to support it (Askins, 1993; Askins et al., 2007; Horn & Koford, 2004; Winter & Faaborg, 1999). However, the central underlying assumption of this assertion is that the surrounding matrix is inhospitable, or at least unused, which has not been directly examined for many species. If a species is able to extend its territory boundaries into the matrix habitat, then it could conceivably occupy a patch smaller than its territory size. Indeed, Schlossberg and King (2008) suggested that the ability of birds to utilize the surrounding matrix might explain their inability to detect significant differences in abundance near edges for certain species. Thus, variation in matrix habitat use may help explain the variation in area‐sensitivity evident among bird communities (Banks‐Leite et al., 2010; Roberts & King, 2017; Shake et al., 2012; Vickery et al., 1994) and possibly other taxa.

The goal of this study was to investigate the extent to which matrix habitat use is related to area‐sensitivity. To accomplish this, we studied two shrubland‐obligate bird species with contrasting area requirements in patches of shrubland habitat within a mature forest matrix. We hypothesized that if that ability to use matrix habitat influences area‐sensitivity, (a) the less area‐sensitive species would display greater use of the mature forest matrix than the more area‐sensitive species, (b) mature forest use would be inversely related to the amount of shrubland habitat available for the less area‐sensitive species, and (c) the frequency of foraging and singing behavior in mature forest would be greater for the less area‐sensitive species.

## 2 METHODS

### 2.1 Study area

This study was conducted within a subset of the forest openings studied by Roberts and King (2017) located within an extensively forested (>90%) area of western Massachusetts (42.5**°**N, −72.3**°**W) in 2014 and 2015. Openings were created through silviculture 4–8 years prior to the study and ranged in size from 0.06–1.01 ha. We focused on two shrubland habitat specialists, the chestnut‐sided warbler (*Setophaga pensylvanica*) and the prairie warbler (*S. discolor*). These species nest almost exclusively in canopy openings created through natural disturbance or silviculture with shrubby regrowth, and are absent from mature forest areas that lack canopy openings. Prairie warblers exhibit pronounced area‐sensitivity, typically occupying forest openings > 1.1 ha, whereas chestnut‐sided warblers regularly occupy considerably smaller openings (<0.27 ha; Roberts & King, 2017). Vegetation within openings consisted of low stature (primarily 0.5–3.5 m tall) saplings including birches (*Betula* spp.), red maple (*Acer rubrum*) and white pine (*Pinus strobus*), and shrubs such as mountain laurel (*Kalmia latifolia*) and brambles (*Rubus* spp.), interspersed with herbaceous vegetation. Openings were separated from each other by mature (~80 yr‐old) deciduous forest buffers (mean width = 43 m) that consisted of red maple (*Acer rubrum*), black birch (*Betula lenta*), American beech (*Fagus grandifolia*), red oak (*Quercus rubra*), eastern hemlock (*Tsuga canadensis*), and white pine (*Pinus strobus*).

### 2.2 Territory mapping and vegetation surveys

Prairie warblers occurred in relatively few openings compared to chestnut‐sided warblers, which occupied the large majority of openings in the study area (Roberts & King, 2017). Therefore, we mapped territories of all prairie warblers within the study area, but mapped chestnut‐sided warblers in a subset of 33 openings. We followed standard mapping methods similar to the spot‐mapping strategy implemented by Streby et al. (2012), who studied a shrubland‐obligate species that occupies relatively comparable habitats. We captured territorial males in mist nets using digital call recordings. We gave each individual a U.S. Geological Survey aluminum band and unique 3‐band color combination. We resighted banded chestnut‐sided warblers and prairie warblers using binoculars (8‐power) from late‐May to early‐July in 2014 and 2015 and recorded all perch locations > 5 m apart during each session using a Global Positioning System (GPS). At each location, we also recorded the behavior(s) of the bird (e.g., singing, foraging, and silent). We mapped birds between sunrise and 1,400 hr, with the majority of bird locations collected between 1,000 and 1,300 hr. We primarily mapped bird movements during this time period due to competing objectives of concurrent projects (Roberts & King, 2017, 2019). To reduce potential temporal bias, observers moved to a different territory after 10 locations were collected in a day (Streby et al., 2012). Observers stopped mapping movements if a bird was lost and could not be relocated within approximately 10 min (Streby et al., 2012). We tried to collect approximately 30 locations per bird. We rotated observers for subsequent visits to each territory in order to reduce bias. We observed no indication that this mapping protocol influenced the behavior or habitat selection of the individual birds studied.

We characterized vegetation within openings at 20 random locations (see Roberts & King, 2017). We determined locations by starting at the center of each opening and walking random distances (1–25 m) at random bearings. We recorded the highest point at which vegetation touched a 3‐m pole placed vertically on the ground, or an upward projection of the pole (for vegetation above 3 m). We recorded the plant species touching the pole at the highest point within four height classes: <0.5 m, 0.5–1.39 m, 1.4–2.9 m, and >2.9 m (Martin et al., 1997). We subsequently grouped plant species into three categories including broad‐leaf vegetation, needle‐leaf vegetation, and forbs, ferns, and grasses.

### 2.3 Estimation of territories and habitat use

We calculated minimum convex polygons (MCP; Mohr, 1947) of bird locations to estimate territory sizes using the “adehabitatHR” package in the R software environment, version 3.1.1 (R Version 3.1.1, http://r‐project.org/). To avoid overestimation of territories, we calculated 95% MCPs, which remove 5% of the most extreme observations located farthest from the centroid of all observations. We assessed the potential impact of mapping effort (i.e, number of GPS locations) on territory size for each species using general linear models. We considered using kernel estimators (Worton, 1987) because they are generally thought to better reflect space use than MCPs (Barg et al., 2005); however, in the relatively small, discrete patches in which this study occurred, 95% and 50% fixed‐kernels (e.g., Lehnen & Rodewald, 2013) buffered bird locations at the edge of openings, such that mature forest was frequently included where use did not occur.

We delineated forest openings satellite imagery and estimated the extent of mature forest and shrubland within each MCP in ArcGIS 10.5.1 (Environmental Systems Research Institute, Redlands, CA, USA). We measured the distance of each bird location within mature forest to the closest forest opening boundary in ArcGIS. Both species regularly used trees on the margins of the openings for singing and other activities, and for this reason, we designated locations within 5 m of the opening boundary, a distance that corresponded to about the radius of the average tree, as within the opening, and locations >5 m from the opening boundary as within mature forest. In several cases (36% of openings) multiple chestnut‐sided warblers occupied a single patch; therefore, we designated the amount of that patch within a bird's territory as the patch size associated with each individual bird for both species in the study. Since resources are exclusively used by territory holders, this designation is reasonable.

### 2.4 Statistical analyses

We used binomial generalized linear models (GLM) with a logit link to analyze proportional response data, including tests of differences between species in overall forest use (proportion of resighted locations in forest), use of forest for foraging, and use of forest for singing, as well as the relationship between patch size and overall forest use. We examined whether chestnut‐sided warblers and prairie warblers differed in relative use of mature forest by including species as a covariates in models. We contrasted use of mature forest for foraging and singing for each species by including species in models of proportion of foraging and singing locations within mature forest. We also used binomial GLMs to examine the importance of vegetation characteristics in predicting overall forest use when patch size was also included in models. We included only one vegetation variable per model to minimize the variable‐sample ratio. Vegetation characteristics included median vegetation height, coefficient of variation (CV) of vegetation height, proportion broad‐leaf plant cover, proportion needle‐leaf plant cover, and proportion forb, fern, and grass cover.

We used a general linear model with an identity link function to relate territory size (ha) to shrubland patch size for each species separately. We use generalized linear models with a gamma distribution and identity link function to relate the amount of forest within territories to shrubland patch size. We fit models and estimated parameters using the “stats” package (R Core Team, 2014) in R. We compared the performance of gamma and gaussian error distributions using Akaike's information criteria corrected for small sample size (AICc; Burnham & Anderson, 2002).

We examined whether each species was more likely to sing or forage in shrubland using one‐sided one‐sample *t* tests (Quinn & Keough, 2003) and a null hypothesis of equal habitat use of forest and openings. We considered relationships to be statistically significant if *p* ≤ .05.

## 3 RESULTS

**Figure 1:**
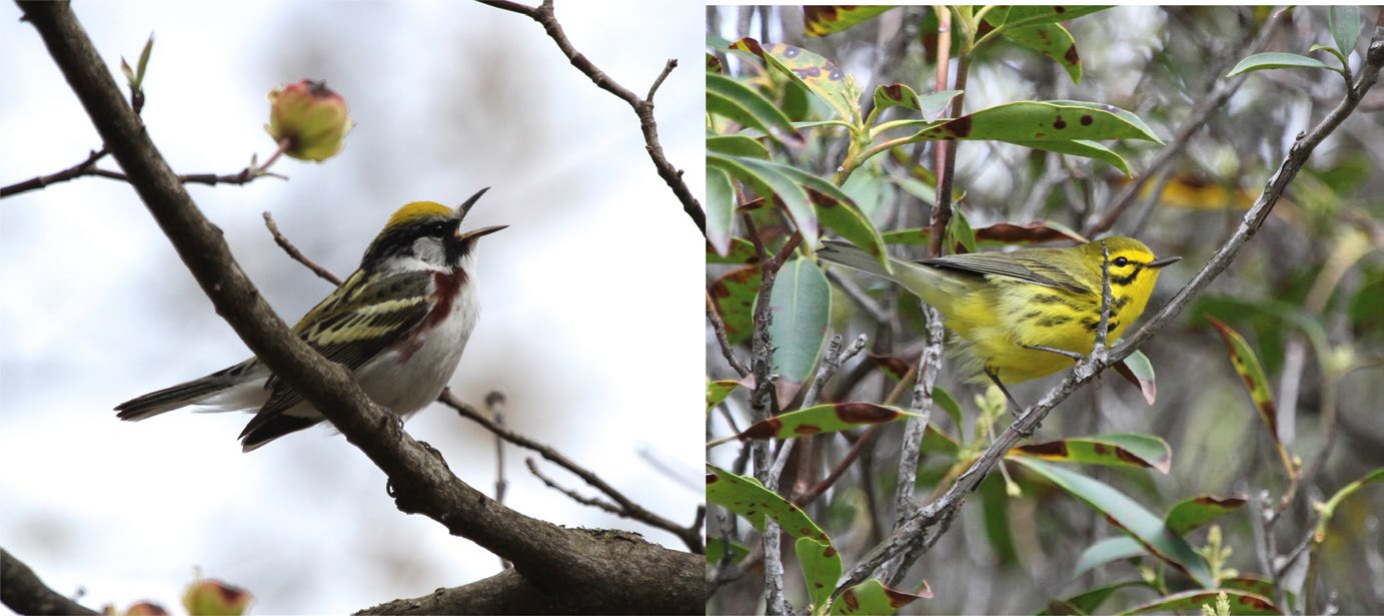
Chestnut‐sided warbler (*Setophaga pensylvanica*; left) and prairie warbler (*S. discolor*; right) in Massachusetts. Photographs by Samuel G. Roberts (used with permission)

**Figure 2:**
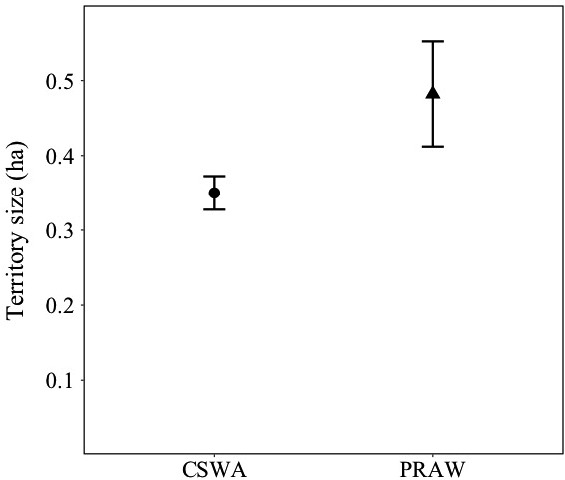
Mean territories sizes for chestnut‐sided warblers (CSWA; *n* = 68) and prairie warblers (PRAW; *n* = 15) in forest canopy openings in western Massachusetts, USA. Error bars depict standard error. Data were collected in 2014 and 2015

We mapped 68 chestnut‐sided warbler (Figure 1) territories and 16 prairie warbler territories across both years of this study. In total, we recorded 2,608 bird resighting locations, averaging 29.3 locations (*SD* = 4.5) per territory. One prairie warbler territory was identified as a potential outlier when examining mature forest use with boxplots, and a review of the data showed that 90% of locations for the bird were in the forest matrix (2.7 *SD* from the mean), which is highly atypical behavior for this species (Akresh et al., 2015). As a result, we removed this bird from subsequent analyses of matrix habitat use. Number of GPS locations did not significantly influence territory size estimates for either species (*p* > .05). Prairie warbler territories (mean = 0.48 ha, *SD* = 0.27) were significantly larger (*β* = 0.144, *SE* = 0.069, *t* = 2.078, *p* = .041) than chestnut‐sided warbler territories (0.35 ha, *SD* = 0.185; Figure 2). Both chestnut‐sided warbler and prairie warbler territory sizes displayed significant positive relationships with patch size (Table 1). Chestnut‐sided warbler MCPs encompassed an average of 45% mature forest and prairie warbler MCPs encompassed an average of 35% mature forest.

**Table 1:** Generalized linear model results for chestnut‐sided warblers (*n* = 68) and prairie warblers (*n* = 15)

Variable		Chestnut‐sided Warbler		Prairie Warbler	
Response	Predictor	*β*	*SE*	*β*	*SE*
Forest area within territory	Patch size^a^	0.277	0.154	0.007	0.486
Proportion of locations in forest	Patch size^a^	−2.592**	0.494	−0.646	0.892
Proportion of foraging locations in forest	Patch size^a^	−1.735*	1.001	1.356`	1.797
Proportion of singing locations in forest	Patch size^a^	−2.564**	0.54	−0.622	1.007
Territory size	Patch size^a^	1.194**	0.154	0.15	0.161

Data was collected in forest openings in western Massachusetts in 2014 and 2015.

^a^ Patch Size = amount (ha) of shrubland habitat within respective territories.

** *p* < .05 and

* *p* < .1.

**Figure 3:**
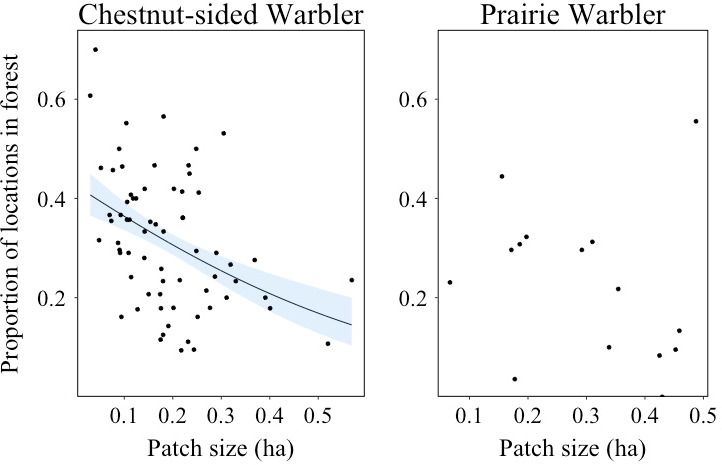
Proportion of bird locations within mature forest in relation to the amount of shrubland habitat within respective territories for chestnut‐sided warblers (*n* = 68) and prairie warblers (*n* = 15). Black line indicates a significant (*p* < .05) trend. Gray region indicates the 95% confidence interval. Data were collected in 2014 and 2015 in western Massachusetts

We observed the majority of locations for both species within forest openings, but chestnut‐sided warblers (mean = 31.6% of locations, *SD* = 0.134) used mature forest significantly more (*β* = −0.353, *SE* = 0.125, *z* = −2.283, *p* = .005) than prairie warblers (mean = 22.9% of locations, *SD* = 0.156). Proportion of locations within mature forest was significantly negatively related to patch size for chestnut‐sided warblers , but not prairie warblers (Table 1; Figure 3).

We observed a significant positive relationship between proportion of chestnut‐sided warbler locations within mature forest and proportion needle‐leaf vegetation and median vegetation height within openings (Table 2). We also observed a significant negative relationship with CV of vegetation height. Prairie warbler showed a significant positive relationship between proportion of locations within mature forest and CV of vegetation height (Table 2). Patch size was significant in all chestnut‐sided warbler vegetation models, but not significant in any prairie warbler vegetation models .

**Table 2:** Generalized linear model results relating proportion of chestnut‐sided warbler (*n* = 68) and prairie warbler (*n* = 15) locations in mature forest to vegetation characteristics. Patch size was included in all models, but coefficients are not presented

Variable	Chestnut‐sided Warbler		Prairie Warbler	
	*β*	*SE*	*β*	*SE*
% forbs, ferns, grasses	−0.276	0.374	−0.69	0.81
% needle‐leaf	0.886**	0.429	−1.384	1.925
% broad‐leaf	−0.61	0.444	1.297	0.883
Vegetation height	0.002**	0.001	−0.002	0.002
CV vegetation height	−0.003**	0.001	0.005**	0.002

Data was collected in forest openings in western Massachusetts in 2014 and 2015.

** *p* < .05.

**Figure 4:**
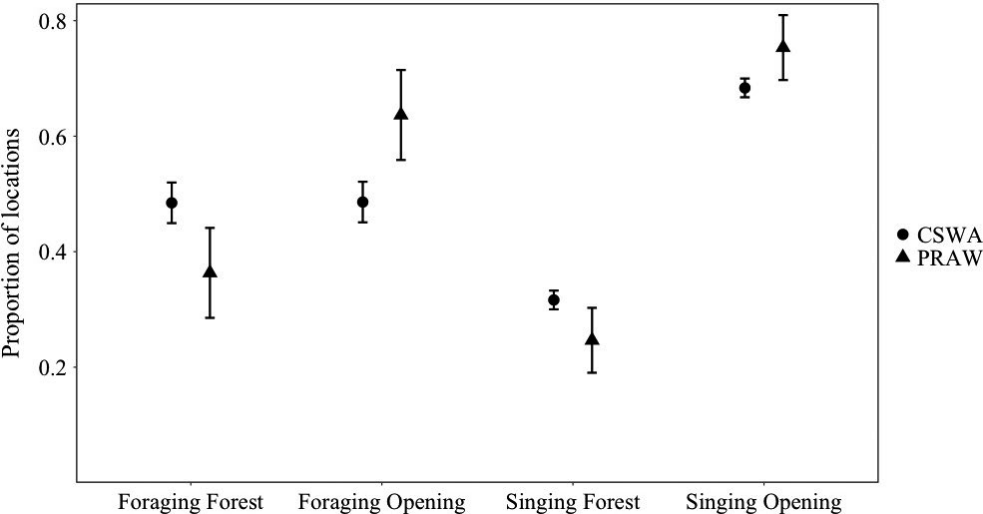
Proportion of foraging and singing locations within mature forest and opening habitat for chestnut‐sided warbler (CSWA; *n* = 68) and prairie warbler (PRAW; *n* = 15). Error bars represent standard error. Data were collected in 2014 and 2015 in western Massachusetts

Chestnut‐sided warblers (*t* = 11.31, *df* = 67, *p* < .001) and prairie warblers (*t* = 6.947, *df* = 15, *p* < .001) sang significantly more within openings compared to mature forest (Figure 4). Chestnut‐sided warblers sang proportionally more often in mature forest than prairie warblers (*β* = −0.321, *SE* = 0.214, *z* = −2.22, *p* = .026). Chestnut‐sided warblers showed a significant negative relationship between proportion of mature forest singing locations and patch size, while prairie warblers displayed no significant relationship (Table 1). Chestnut‐sided warblers foraged proportionally more often (*β* = −0.914, *SE* = 0.243, *z* = −3.75, *p* < .01) in surrounding mature forest (mean = 48.4% of foraging locations, *σ* = 0.29) than prairie warblers (mean = 32.9% of foraging locations, *SD* = 0.291). Prairie warblers were significantly more likely to forage in openings (*t* = 2.265, *df* = 14, *p* = .019), whereas chestnut‐sided warblers showed no preference in habitat for foraging (*t* = −0.398, *df* = 67, *p* = .65). Neither chestnut‐sided warblers nor prairie warblers showed a significant relationship between the proportion of foraging observations within mature forest and patch size (Table 1).

## 4 DISCUSSION

Despite documentation of area‐sensitivity in North American passerines (e.g., Freemark & Collins, 1992; Robbins et al., 1989; Roberts & King, 2017; Shake et al., 2012; Winter et al., 2006) and other taxa (Bender et al., 1998; Pe’er et al., 2013), the underlying mechanisms that determine this phenomenon have remained elusive (Lehnen & Rodewald, 2009; Ribic et al., 2009). The findings of this study suggest that relative area‐sensitivity may in part reflect the ability (or lack thereof) of individual species to utilize the surrounding matrix. Chestnut‐sided warblers, which are relatively insensitive to patch area and frequently occupy forest canopy openings <0.27 ha, used surrounding mature forest matrix significantly more than prairie warblers, which are highly sensitive and typically occupy openings >1.1 ha; Roberts & King, 2017). Chestnut‐sided warblers also increased their use of the mature forest matrix as patch size decreased, whereas prairie warblers showed no such relationship. These findings suggest that not only does the less area‐sensitive chestnut‐sided warbler appear to have a greater capacity for mature forest use than its area‐sensitive counterpart, but they can accommodate small patch sizes by increasing use of adjacent habitat. Overall, prairie warblers, and other area‐sensitive species, may require larger habitat patches in part because they are less capable of utilizing the surrounding matrix.

This study provides support for the hypothesis of Schlossberg and King (2008) that patterns in relative abundance near edges may be determined by the degree to which species can use the surrounding matrix—species for which abundance does not decrease significantly near edges may make greater use of the surrounding matrix and vice versa. This is further supported by the general pattern within the shrubland bird community of minimal area‐sensitivity (occupy openings ≤ 0.27 ha; Roberts & King, 2017) for species that do not decline in abundance near edges such as chestnut‐sided warbler, common yellowthroat (*Geothlypis trichas*), eastern towhee (*Pipilo erythrophthalmus*), gray catbird (*Dumatella carolinensis*), and black‐and‐white warbler (*Mniotilta varia;* Schlossberg & King, 2008). In contrast, species that are significantly less abundant near edges, such as indigo bunting (*Passerina cyanea*), prairie warbler, and yellow‐breasted chat (Rodewald and Vitz, Lehnen & Rodewald, 2013; Schlossberg & King, 2008), exhibit greater area requirements (0.56–2.3 ha; Roberts & King, 2017; Shake et al., 2012). This pattern supports the generality of the relationship between matrix use and area‐sensitivity in shrubland birds.

Our examination of foraging behavior suggests that prairie warblers could be limited to larger openings due to an inability to supplement food deficiencies using mature forest when available shrubland habitat is limited. Prairie warblers foraged significantly more often within openings than mature forest, while chestnut‐sided warblers showed no clear preference for foraging habitat and foraged slightly more often in mature forest than in openings overall. Moreover, chestnut‐sided warblers showed a tendency (albeit nonsignificant) to forage more often within mature forest when the patch size was smaller. Food abundance has been shown to be a natural limiting factor in small patches of habitat for other groups of birds (e.g., Burke & Nol, 1998; Zanette et al., 2000), but Champlin et al. (2009) found no difference in bird use of small forest openings (0.13–0.5 ha) when food resources were experimentally reduced. However, if less area‐sensitive species are more capable of using matrix habitat, it is possible that Champlin et al. (2009) failed to observe a decline in bird use because the species that occupied the openings in their study were able to use the surrounding mature forest as a supplemental resource.

Apart from extent of available habitat, vegetative characteristics such as species composition and structure may also influence relative food abundance or availability in forest openings. Most passerines rely on arthropods as their primary food resource during the breeding season (Rodewald & Vitz, 2005) and taxa such as Lepidopterans (e.g., Bubova et al., 2015; Poyry et al., 2006) and Hymenopterans (Roberts, King, & Milam, 2017) have been shown to be negatively impacted by succession in early‐successional habitats. Vegetative structure has also been shown to impact food availability for birds (Holmes & Schultz, 1988). Therefore, reduced food abundance or ability to effectively acquire food in taller early‐successional vegetation may explain the observed positive association between mature forest use by chestnut‐sided warblers and vegetation height within openings. Similarly, the observed positive and negative relationships with needle‐leaf cover and CV vegetation height respectively may reflect the relative availability of invertebrate prey items that these factors confer. Notably, patch size was significant in all vegetation models for chestnut‐sided warbler, indicating that the amount of shrubland available still influenced mature forest use even when the effects of vegetation were accounted for.

Chestnut‐sided warblers displayed a greater tendency to incorporate mature forest habitat into the area they actively defend (via singing). Both species broadcasted their songs more often from within openings, but chestnut‐sided warblers were significantly more likely to sing within mature forest than prairie warblers, and the proportion of singing locations within mature forest increased significantly as patch size decreased, whereas prairie warbler singing within mature forest stayed consistent overall irrespective of patch size. This further indicates that area‐sensitive shrubland species are more likely to restrict their territories to shrubland habitats than their less area‐sensitive counterparts.

Although our finding that the more area‐sensitive species was less inclined to use the matrix habitat supports the notion that territory size imposes a lower boundary on patch sizes that are usable by a species (Askins, 1993; Askins et al., 2007), both species incorporated at least some mature forest into territories, as do other shrubland‐obligate species (Streby et al., 2012), illustrating that territory size is not necessarily a reliable measure of specific area requirements. Moreover, both chestnut‐sided warblers and prairie warblers exhibited a wide range of territory sizes (0.12–1 ha and 0.18–1.3 ha, respectively), which were positively associated with patch size. This suggests that researchers should take care when using generalized territory size estimates to assess habitat suitability and inform management recommendations.

Despite increased forest use by chestnut‐sided warblers with less available shrubland, both territory size and the amount of mature forest within territories were not significantly related to shrubland patch size. This was counter to our expectation that, as patch size decreased, individuals would need to extend their territories into the surrounding matrix in order to maintain their standard territory size (0.4–1.1 ha [Byers et al., 2013]) and compensate for reduced food resources. Since territory size is generally inversely related to food abundance for birds and other taxa (e.g., Marshall & Cooper, 2004; Simon, 1975; Smith & Shugart, 1987; Stenger, 1958), this pattern suggests that, if the matrix was primarily used for foraging, adjacent forest canopies provided enough resources such that most birds did not have to expand their territories very far into the forest to find a sufficient abundance of prey.

Our findings suggest that matrix habitat use plays at least a partial role in determining the relative area‐sensitivity of shrubland‐obligate passerines and possibly other taxa. Increased understanding of the drivers of habitat use in isolated habitats is key to developing comprehensive conservation strategies for area‐sensitive species. Although our observations are consistent with our hypothesis that matrix habitat use by songbirds in this system influences their sensitivity to patch area, area‐sensitivity is likely driven by a potential suite of correlated factors (Bayard & Elphick, 2010). Thus, we concede that some unmeasured variable or variables may have caused the patterns of patch occupancy we observed. Future research should consider experimental manipulation or attempt to minimize the correlation of potential drivers of area‐sensitivity through careful study design. Caution should be exercised when considering these findings within the context of other taxa and future research is needed before broad generalizations can be made. Since area‐sensitivity appears to be related to matrix habitat use, species‐area relationships are likely dynamic, varying by matrix type, landscape context, and other potential factors (Ockinger et al., 2012; Pe’er et al., 2013; Prugh et al., 2008). For example, chestnut‐sided warblers readily utilized the surrounding mature forest in this study, but may be less likely to utilize the matrix when situated within extensive grassland, agriculture, or other anthropogenic habitats due to species‐specific foraging strategies (e.g., Holmes & Schultz, 1988) or other life‐history traits (Ockinger et al., 2010). Thus, species may display varying patch area relationships depending upon the type of matrix present.

## CONFLICT OF INTEREST

None declared.

## AUTHOR CONTRIBUTION

**H. Patrick Roberts:** Conceptualization (equal); Data curation (lead); Formal analysis (equal); Investigation (equal); Methodology (equal); Writing‐original draft (lead); Writing‐review & editing (lead). **David I. King:** Conceptualization (equal); Formal analysis (supporting); Funding acquisition (lead); Investigation (equal); Project administration (equal); Supervision (lead); Writing‐original draft (supporting); Writing‐review & editing (supporting).

## Data Availability

The data used in this study are archived in the Dryad data repository: https://doi.org/10.5061/dryad.tqjq2bvx1.
